# 1-Cyclo­hexyl-5-(4-meth­oxy­phen­yl)-1*H*-pyrazole-4-carb­oxy­lic acid

**DOI:** 10.1107/S1600536811050872

**Published:** 2011-11-30

**Authors:** Hoong-Kun Fun, Ching Kheng Quah, B. Chandrakantha, A. M. Isloor, Prakash Shetty

**Affiliations:** a X-ray Crystallography Unit, School of Physics, Universiti Sains Malaysia, 11800 USM, Penang, Malaysia; b Department of Chemistry, Manipal Institute of Technology, Manipal 576 104, India; c Medicinal Chemistry Division, Department of Chemistry, National Institute of Technology-Karnataka, Surathkal, Mangalore 575 025, India; d Department of Printing, Manipal Institute of Technology, Manipal 576 104, India

## Abstract

In the title compound, C_17_H_20_N_2_O_3_, the meth­oxy­phenyl unit is disordered over two sets of sites in a 0.715 (4):0.285 (4) ratio. The pyrazole ring forms dihedral angles of 55.88 (16) and 72.6 (4)° with the benzene rings of its major and minor components, respectively. The cyclo­hexane ring adopts a chair conformation and its C—N bond is in an equatorial orientation. In the crystal, mol­ecules are linked into inversion dimers by pairs of O—H⋯O hydrogen bonds, generating *R*_2_^2^(8) loops.

## Related literature

For bond-length data, see: Allen *et al.* (1987 ▶). For related structures and medicinal background to pyrazole derivatives, see: Fun *et al.* (2010*a* ▶,*b* ▶, 2011 ▶). For hydrogen-bond motifs, see: Bernstein *et al.* (1995 ▶). For ring conformations, see: Cremer & Pople (1975 ▶).
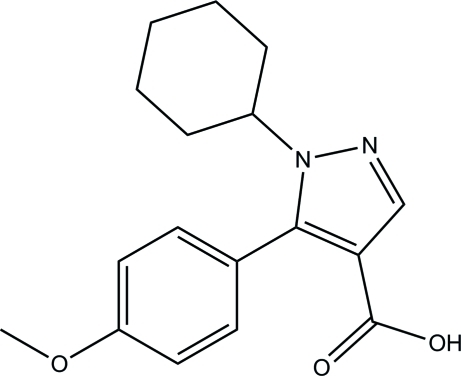


## Experimental

### 

#### Crystal data

C_17_H_20_N_2_O_3_
*M* *_r_* = 300.35
Monoclinic, 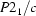

*a* = 12.0722 (9) Å
*b* = 12.7180 (9) Å
*c* = 11.7739 (8) Å
β = 118.698 (1)°
*V* = 1585.6 (2) Å^3^
*Z* = 4
Mo *K*α radiation
μ = 0.09 mm^−1^
*T* = 296 K
0.39 × 0.20 × 0.15 mm

#### Data collection

Bruker SMART APEXII DUO CCD diffractometer
Absorption correction: multi-scan (*SADABS*; Bruker, 2009 ▶) *T*_min_ = 0.967, *T*_max_ = 0.987
24474 measured reflections
4585 independent reflections
2766 reflections with *I* > 2σ(*I*)
*R*_int_ = 0.049

#### Refinement

*R*[*F* ^2^ > 2σ(*F* ^2^)] = 0.056
*wR*(*F* ^2^) = 0.159
*S* = 1.03
4585 reflections
239 parameters
17 restraints
H-atom parameters constrained
Δρ_max_ = 0.21 e Å^−3^
Δρ_min_ = −0.28 e Å^−3^

### 

Data collection: *APEX2* (Bruker, 2009 ▶); cell refinement: *SAINT* (Bruker, 2009 ▶); data reduction: *SAINT*; program(s) used to solve structure: *SHELXTL* (Sheldrick, 2008 ▶); program(s) used to refine structure: *SHELXTL*; molecular graphics: *SHELXTL*; software used to prepare material for publication: *SHELXTL* and *PLATON* (Spek, 2009 ▶).

## Supplementary Material

Crystal structure: contains datablock(s) global, I. DOI: 10.1107/S1600536811050872/hb6534sup1.cif

Structure factors: contains datablock(s) I. DOI: 10.1107/S1600536811050872/hb6534Isup2.hkl

Supplementary material file. DOI: 10.1107/S1600536811050872/hb6534Isup3.cml

Additional supplementary materials:  crystallographic information; 3D view; checkCIF report

## Figures and Tables

**Table 1:** Hydrogen-bond geometry (Å, °)

*D*—H⋯*A*	*D*—H	H⋯*A*	*D*⋯*A*	*D*—H⋯*A*
O2—H1*O*2⋯O1^i^	0.91	1.73	2.640 (2)	174

Symmetry code: (i) 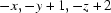.

**Fig. 1.**
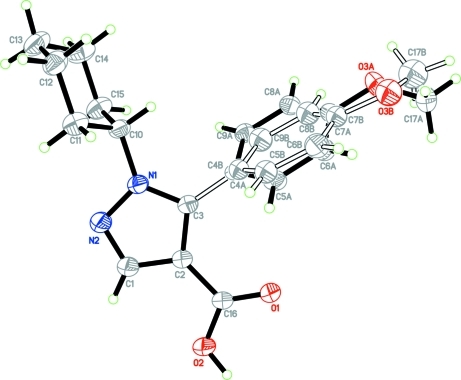
The molecular structure of the title compound showing 30% probability displacement ellipsoids for non-H atoms. Both major and minor components of disorder are shown.

**Fig. 2.**
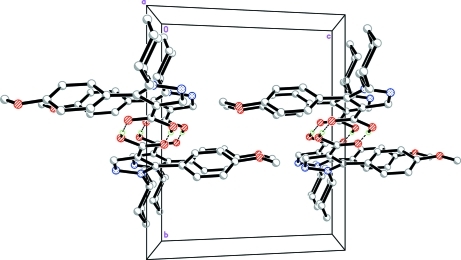
The crystal structure of the major component of the title compound, viewed along the a axis. H atoms not involved in hydrogen bonds (dashed lines) have been omitted for clarity.

## supplementary crystallographic information

### Comment

As part of our ongoing structural studies of pyrazole derivatives (Fun *et al.*, 2010*a*, 2010*b*), we now describe the synthesis and structure of the title compound, (I).

The molecular structure is shown in Fig. 1. The methoxy phenyl moiety (O3/C4-C9/C17) is disordered over two sets of sites with refined site occupancies of 0.715 (4): 0.285 (4). The pyrazole ring (N1/N2/C1-C3) forms dihedral angles of 55.88 (16) and 72.6 (4)° with the benzene rings (C4-C9) of major and minor components of the methoxy phenyl moiety, respectively. Bond lengths (Allen *et al.*, 1987) and angles are within normal ranges and are comparable to related structures (Fun *et al.*, 2010*a*, 2010*b*, 2011). The cyclohexane ring (C10-C15) adopts a chair conformation with puckering parameters (Cremer & Pople, 1975) Q = 0.571 (2) Å, Θ = 1.0 (2)° and φ = 300 (19)°.

In the crystal (Fig. 2), molecules are linked into inversion dimers by pairs of O2–H1O2···O1 hydrogen bonds (Table 1), generating eight-membered R^2^_2_(8) ring motifs (Bernstein *et al.*, 1995).

### Experimental

A mixture of ethyl-3-(dimethylamino)-2-(4-methoxy phenylcarbonyl) prop-2-enoate (2.0g, 0.0088 mol) and cyclohexyl hydrazine (1.0 g, 0.0096 mol) in absolute ethanol (20 ml) was refluxed for 2h. On cooling, the separated colorless needle-shaped crystals of 5-(4-methoxy phenyl)-1-phenyl-1*H*-pyrazole-4- carboxylic acid ethyl ester were collected by filtration (yield: 2.0 g, 86%, *m.p.*: 390-395K). To a stirred solution of ester (1.0 g, 0.0031 mol) in THF with water (1:1, 20 ml) was added lithium hydroxide (0.26 g, 0.0062 mol) and the mixture was stirred at RT for 6h. The reaction mixture was concentrated and acidified with 10% citric acid solution. The solid that separated out was filtered and dried under high vacuum to afford title compound as colorless crystalline solid. Compound was recrystallized from methanol to yield colourless needles (yield: 1.5 g, 83%, *m.p.* 413-418K).

### Refinement

Atom H1O2 was located in a difference Fourier map and refined using a riding model with O2-H1O2 = 0.9133 Å and *U*_iso_(H) = 1.5 *U*_eq_(O). The remaining H atoms were positioned geometrically and refined using a riding model with C–H = 0.93-0.98 Å and *U*_iso_(H) = 1.2 or 1.5 *U*_eq_(C). A rotating-group model was applied for the methyl groups. The methoxy phenyl moiety (O3/C4-C9/C17) is disordered over two positions with refined site occupancies of 0.715 (4): 0.285 (4).

### Crystal data

**Table:**

C_17_H_20_N_2_O_3_	*F*(000) = 640
*M**_r_* = 300.35	*D*_x_ = 1.258 Mg m^−^^3^
Monoclinic, *P*2_1_/*c*	Mo *K*α radiation, λ = 0.71073 Å
Hall symbol: -P 2ybc	Cell parameters from 5000 reflections
*a* = 12.0722 (9) Å	θ = 2.5–29.8°
*b* = 12.7180 (9) Å	µ = 0.09 mm^−^^1^
*c* = 11.7739 (8) Å	*T* = 296 K
β = 118.698 (1)°	Needle, colourless
*V* = 1585.6 (2) Å^3^	0.39 × 0.20 × 0.15 mm
*Z* = 4	

### Data collection

**Table:**

Bruker SMART APEXII DUO CCD diffractometer	4585 independent reflections
Radiation source: fine-focus sealed tube	2766 reflections with *I* > 2σ(*I*)
graphite	*R*_int_ = 0.049
φ and ω scans	θ_max_ = 29.9°, θ_min_ = 1.9°
Absorption correction: multi-scan (*SADABS*; Bruker, 2009)	*h* = −16→16
*T*_min_ = 0.967, *T*_max_ = 0.987	*k* = −17→17
24474 measured reflections	*l* = −16→16

### Refinement

**Table:**

Refinement on *F*^2^	Primary atom site location: structure-invariant direct methods
Least-squares matrix: full	Secondary atom site location: difference Fourier map
*R*[*F*^2^ > 2σ(*F*^2^)] = 0.056	Hydrogen site location: inferred from neighbouring sites
*wR*(*F*^2^) = 0.159	H-atom parameters constrained
*S* = 1.03	*w* = 1/[σ^2^(*F*_o_^2^) + (0.0614*P*)^2^ + 0.3536*P*] where *P* = (*F*_o_^2^ + 2*F*_c_^2^)/3
4585 reflections	(Δ/σ)_max_ = 0.001
239 parameters	Δρ_max_ = 0.21 e Å^−^^3^
17 restraints	Δρ_min_ = −0.28 e Å^−^^3^

### Special details

**Table:**

Geometry. All esds (except the esd in the dihedral angle between two l.s. planes) are estimated using the full covariance matrix. The cell esds are taken into account individually in the estimation of esds in distances, angles and torsion angles; correlations between esds in cell parameters are only used when they are defined by crystal symmetry. An approximate (isotropic) treatment of cell esds is used for estimating esds involving l.s. planes.
Refinement. Refinement of F^2^ against ALL reflections. The weighted R-factor wR and goodness of fit S are based on F^2^, conventional R-factors R are based on F, with F set to zero for negative F^2^. The threshold expression of F^2^ > 2sigma(F^2^) is used only for calculating R-factors(gt) etc. and is not relevant to the choice of reflections for refinement. R-factors based on F^2^ are statistically about twice as large as those based on F, and R- factors based on ALL data will be even larger.

### Fractional atomic coordinates and isotropic or equivalent isotropic displacement parameters (Å^2^)

**Table:**

	*x*	*y*	*z*	*U*_iso_*/*U*_eq_	Occ. (<1)
O1	0.07405 (12)	0.45236 (14)	0.92263 (12)	0.0870 (5)	
O2	0.15254 (12)	0.47797 (13)	1.13352 (11)	0.0814 (5)	
H1O2	0.0749	0.4999	1.1195	0.122*	
O3A	0.1055 (2)	0.39532 (19)	0.44671 (17)	0.0769 (8)	0.715 (4)
C4A	0.2726 (5)	0.3627 (5)	0.8457 (5)	0.0409 (10)	0.715 (4)
C5A	0.1549 (3)	0.3174 (3)	0.7674 (3)	0.0528 (7)	0.715 (4)
H5AA	0.1137	0.2823	0.8057	0.063*	0.715 (4)
C6A	0.0978 (4)	0.3237 (3)	0.6330 (3)	0.0573 (9)	0.715 (4)
H6AA	0.0206	0.2909	0.5820	0.069*	0.715 (4)
C7A	0.1563 (3)	0.3787 (3)	0.5760 (2)	0.0489 (6)	0.715 (4)
C8A	0.2749 (3)	0.4202 (2)	0.6523 (3)	0.0523 (7)	0.715 (4)
H8AA	0.3168	0.4540	0.6139	0.063*	0.715 (4)
C9A	0.3314 (3)	0.4121 (2)	0.7843 (3)	0.0453 (6)	0.715 (4)
H9AA	0.4115	0.4405	0.8341	0.054*	0.715 (4)
C17A	−0.0253 (3)	0.3757 (3)	0.3667 (3)	0.0861 (11)	0.715 (4)
H17A	−0.0504	0.3991	0.2802	0.129*	0.715 (4)
H17B	−0.0727	0.4129	0.3999	0.129*	0.715 (4)
H17C	−0.0413	0.3016	0.3657	0.129*	0.715 (4)
O3B	0.0427 (5)	0.3332 (4)	0.4351 (5)	0.0707 (18)*	0.285 (4)
C4B	0.2589 (16)	0.3476 (14)	0.8323 (13)	0.043 (3)*	0.285 (4)
C5B	0.1646 (8)	0.2768 (6)	0.7716 (8)	0.051 (2)*	0.285 (4)
H5B	0.1475	0.2291	0.8211	0.061*	0.285 (4)
C6	0.0940 (9)	0.2735 (8)	0.6402 (9)	0.059 (2)	0.285 (4)
H6B	0.0277	0.2259	0.6006	0.070*	0.285 (4)
C7B	0.1219 (7)	0.3407 (6)	0.5680 (7)	0.0456 (18)*	0.285 (4)
C8B	0.2195 (9)	0.4113 (7)	0.6240 (8)	0.060 (2)*	0.285 (4)
H8B	0.2389	0.4551	0.5727	0.072*	0.285 (4)
C9B	0.2888 (8)	0.4168 (8)	0.7578 (9)	0.062 (3)*	0.285 (4)
H9B	0.3539	0.4654	0.7976	0.074*	0.285 (4)
C17B	0.0494 (10)	0.4149 (7)	0.3569 (10)	0.092 (3)*	0.285 (4)
H17D	−0.0130	0.4034	0.2684	0.138*	0.285 (4)
H17E	0.1319	0.4157	0.3637	0.138*	0.285 (4)
H17F	0.0338	0.4812	0.3857	0.138*	0.285 (4)
N1	0.44673 (12)	0.32694 (11)	1.06351 (12)	0.0524 (3)	
N2	0.48284 (13)	0.34101 (14)	1.19163 (13)	0.0676 (4)	
C1	0.38353 (16)	0.38444 (16)	1.19118 (16)	0.0650 (5)	
H1A	0.3804	0.4035	1.2658	0.078*	
C2	0.28347 (14)	0.39865 (14)	1.06610 (15)	0.0525 (4)	
C3	0.32756 (13)	0.36065 (13)	0.98426 (14)	0.0460 (3)	
C10	0.53239 (13)	0.27005 (13)	1.02944 (15)	0.0482 (4)	
H10A	0.4887	0.2600	0.9354	0.058*	
C11	0.56252 (17)	0.16218 (14)	1.09219 (18)	0.0602 (4)	
H11A	0.4849	0.1227	1.0639	0.072*	
H11B	0.6021	0.1698	1.1855	0.072*	
C12	0.65043 (16)	0.10250 (14)	1.05635 (18)	0.0620 (5)	
H12A	0.6723	0.0355	1.1013	0.074*	
H12B	0.6072	0.0884	0.9640	0.074*	
C13	0.76923 (16)	0.16370 (16)	1.0912 (2)	0.0734 (6)	
H13A	0.8210	0.1254	1.0628	0.088*	
H13B	0.8171	0.1715	1.1845	0.088*	
C14	0.73834 (17)	0.27110 (16)	1.0285 (2)	0.0730 (6)	
H14A	0.6976	0.2632	0.9351	0.088*	
H14B	0.8159	0.3103	1.0555	0.088*	
C15	0.65178 (16)	0.33218 (14)	1.0652 (2)	0.0622 (5)	
H15A	0.6952	0.3460	1.1575	0.075*	
H15B	0.6302	0.3992	1.0203	0.075*	
C16	0.16128 (15)	0.44458 (16)	1.03370 (16)	0.0589 (4)	

### Atomic displacement parameters (Å^2^)

**Table:**

	*U*^11^	*U*^22^	*U*^33^	*U*^12^	*U*^13^	*U*^23^
O1	0.0572 (7)	0.1573 (15)	0.0477 (7)	0.0425 (8)	0.0262 (6)	0.0055 (8)
O2	0.0613 (7)	0.1362 (13)	0.0527 (7)	0.0334 (8)	0.0323 (6)	−0.0050 (7)
O3A	0.0761 (14)	0.1104 (18)	0.0383 (10)	0.0057 (13)	0.0227 (9)	0.0049 (10)
C4A	0.0356 (19)	0.051 (2)	0.0423 (17)	0.0014 (13)	0.0234 (14)	−0.0032 (16)
C5A	0.0468 (14)	0.068 (2)	0.0531 (15)	−0.0086 (15)	0.0315 (12)	−0.0019 (14)
C6A	0.0447 (14)	0.077 (2)	0.0497 (16)	−0.0107 (18)	0.0226 (12)	−0.0097 (17)
C7A	0.0523 (16)	0.0575 (17)	0.0399 (13)	0.0057 (14)	0.0246 (12)	0.0014 (12)
C8A	0.0507 (16)	0.0666 (16)	0.0485 (14)	0.0042 (13)	0.0308 (13)	0.0101 (11)
C9A	0.0399 (14)	0.0515 (14)	0.0479 (14)	0.0012 (12)	0.0237 (12)	0.0039 (10)
C17A	0.0647 (18)	0.121 (3)	0.0512 (16)	0.0264 (19)	0.0110 (14)	−0.0099 (16)
C6	0.047 (4)	0.073 (6)	0.059 (4)	−0.013 (4)	0.027 (3)	−0.003 (4)
N1	0.0429 (7)	0.0684 (9)	0.0456 (7)	0.0084 (6)	0.0212 (6)	−0.0078 (6)
N2	0.0543 (8)	0.0971 (12)	0.0453 (8)	0.0182 (8)	0.0190 (6)	−0.0117 (7)
C1	0.0571 (10)	0.0919 (14)	0.0469 (9)	0.0174 (9)	0.0257 (8)	−0.0064 (9)
C2	0.0466 (8)	0.0704 (10)	0.0457 (8)	0.0107 (7)	0.0261 (7)	0.0011 (7)
C3	0.0401 (7)	0.0557 (9)	0.0461 (8)	0.0029 (6)	0.0238 (6)	0.0001 (6)
C10	0.0380 (7)	0.0575 (9)	0.0505 (8)	0.0035 (6)	0.0224 (6)	−0.0086 (7)
C11	0.0566 (9)	0.0603 (10)	0.0690 (11)	0.0006 (8)	0.0344 (9)	−0.0028 (8)
C12	0.0566 (10)	0.0564 (10)	0.0689 (11)	0.0086 (8)	0.0268 (9)	−0.0059 (8)
C13	0.0450 (9)	0.0802 (13)	0.0877 (14)	0.0097 (9)	0.0259 (9)	−0.0166 (11)
C14	0.0508 (9)	0.0802 (13)	0.1005 (15)	−0.0074 (9)	0.0463 (10)	−0.0130 (11)
C15	0.0515 (9)	0.0560 (10)	0.0826 (13)	−0.0032 (7)	0.0351 (9)	−0.0106 (9)
C16	0.0501 (9)	0.0872 (13)	0.0474 (9)	0.0161 (8)	0.0297 (8)	0.0036 (8)

### Geometric parameters (Å, °)

**Table:**

O1—C16	1.227 (2)	C8B—H8B	0.9300
O2—C16	1.3007 (19)	C9B—H9B	0.9300
O2—H1O2	0.9133	C17B—H17D	0.9600
O3A—C7A	1.357 (3)	C17B—H17E	0.9600
O3A—C17A	1.419 (4)	C17B—H17F	0.9600
C4A—C9A	1.384 (4)	N1—C3	1.3546 (19)
C4A—C5A	1.394 (5)	N1—N2	1.3660 (18)
C4A—C3	1.436 (5)	N1—C10	1.4671 (18)
C5A—C6A	1.393 (4)	N2—C1	1.318 (2)
C5A—H5AA	0.9300	C1—C2	1.397 (2)
C6A—C7A	1.376 (4)	C1—H1A	0.9300
C6A—H6AA	0.9300	C2—C3	1.392 (2)
C7A—C8A	1.379 (4)	C2—C16	1.457 (2)
C8A—C9A	1.368 (4)	C10—C15	1.515 (2)
C8A—H8AA	0.9300	C10—C11	1.517 (2)
C9A—H9AA	0.9300	C10—H10A	0.9800
C17A—H17A	0.9600	C11—C12	1.520 (2)
C17A—H17B	0.9600	C11—H11A	0.9700
C17A—H17C	0.9600	C11—H11B	0.9700
O3B—C7B	1.391 (8)	C12—C13	1.505 (3)
O3B—C17B	1.416 (10)	C12—H12A	0.9700
C4B—C5B	1.355 (12)	C12—H12B	0.9700
C4B—C9B	1.407 (13)	C13—C14	1.512 (3)
C4B—C3	1.578 (14)	C13—H13A	0.9700
C5B—C6	1.363 (10)	C13—H13B	0.9700
C5B—H5B	0.9300	C14—C15	1.521 (2)
C6—C7B	1.358 (10)	C14—H14A	0.9700
C6—H6B	0.9300	C14—H14B	0.9700
C7B—C8B	1.373 (9)	C15—H15A	0.9700
C8B—C9B	1.386 (10)	C15—H15B	0.9700
			
C16—O2—H1O2	117.2	N2—C1—H1A	123.7
C7A—O3A—C17A	118.5 (3)	C2—C1—H1A	123.7
C9A—C4A—C5A	117.3 (4)	C3—C2—C1	105.08 (14)
C9A—C4A—C3	121.9 (4)	C3—C2—C16	129.32 (15)
C5A—C4A—C3	120.8 (3)	C1—C2—C16	125.60 (15)
C6A—C5A—C4A	121.2 (3)	N1—C3—C2	105.40 (13)
C6A—C5A—H5AA	119.4	N1—C3—C4A	123.2 (3)
C4A—C5A—H5AA	119.4	C2—C3—C4A	131.2 (3)
C7A—C6A—C5A	119.6 (3)	N1—C3—C4B	123.6 (8)
C7A—C6A—H6AA	120.2	C2—C3—C4B	130.8 (8)
C5A—C6A—H6AA	120.2	N1—C10—C15	111.61 (13)
O3A—C7A—C6A	124.4 (3)	N1—C10—C11	110.39 (13)
O3A—C7A—C8A	116.1 (3)	C15—C10—C11	111.26 (13)
C6A—C7A—C8A	119.5 (2)	N1—C10—H10A	107.8
C9A—C8A—C7A	120.4 (2)	C15—C10—H10A	107.8
C9A—C8A—H8AA	119.8	C11—C10—H10A	107.8
C7A—C8A—H8AA	119.8	C10—C11—C12	110.64 (15)
C8A—C9A—C4A	121.8 (3)	C10—C11—H11A	109.5
C8A—C9A—H9AA	119.1	C12—C11—H11A	109.5
C4A—C9A—H9AA	119.1	C10—C11—H11B	109.5
C7B—O3B—C17B	117.3 (7)	C12—C11—H11B	109.5
C5B—C4B—C9B	119.3 (11)	H11A—C11—H11B	108.1
C5B—C4B—C3	121.2 (9)	C13—C12—C11	111.52 (15)
C9B—C4B—C3	119.3 (9)	C13—C12—H12A	109.3
C4B—C5B—C6	121.9 (9)	C11—C12—H12A	109.3
C4B—C5B—H5B	119.0	C13—C12—H12B	109.3
C6—C5B—H5B	119.0	C11—C12—H12B	109.3
C7B—C6—C5B	118.9 (8)	H12A—C12—H12B	108.0
C7B—C6—H6B	120.5	C12—C13—C14	110.78 (15)
C5B—C6—H6B	120.5	C12—C13—H13A	109.5
C6—C7B—C8B	121.7 (7)	C14—C13—H13A	109.5
C6—C7B—O3B	114.7 (7)	C12—C13—H13B	109.5
C8B—C7B—O3B	123.6 (7)	C14—C13—H13B	109.5
C7B—C8B—C9B	119.3 (8)	H13A—C13—H13B	108.1
C7B—C8B—H8B	120.3	C13—C14—C15	111.37 (17)
C9B—C8B—H8B	120.3	C13—C14—H14A	109.4
C8B—C9B—C4B	118.8 (9)	C15—C14—H14A	109.4
C8B—C9B—H9B	120.6	C13—C14—H14B	109.4
C4B—C9B—H9B	120.6	C15—C14—H14B	109.4
O3B—C17B—H17D	109.5	H14A—C14—H14B	108.0
O3B—C17B—H17E	109.5	C10—C15—C14	110.26 (14)
H17D—C17B—H17E	109.5	C10—C15—H15A	109.6
O3B—C17B—H17F	109.5	C14—C15—H15A	109.6
H17D—C17B—H17F	109.5	C10—C15—H15B	109.6
H17E—C17B—H17F	109.5	C14—C15—H15B	109.6
C3—N1—N2	112.92 (12)	H15A—C15—H15B	108.1
C3—N1—C10	128.51 (13)	O1—C16—O2	122.41 (15)
N2—N1—C10	118.26 (12)	O1—C16—C2	123.66 (15)
C1—N2—N1	104.07 (13)	O2—C16—C2	113.93 (14)
N2—C1—C2	112.52 (15)		
			
C9A—C4A—C5A—C6A	−1.4 (8)	C1—C2—C3—N1	0.32 (19)
C3—C4A—C5A—C6A	176.6 (4)	C16—C2—C3—N1	179.92 (18)
C4A—C5A—C6A—C7A	−2.3 (6)	C1—C2—C3—C4A	−173.6 (3)
C17A—O3A—C7A—C6A	14.3 (4)	C16—C2—C3—C4A	6.0 (4)
C17A—O3A—C7A—C8A	−166.1 (3)	C1—C2—C3—C4B	175.8 (7)
C5A—C6A—C7A—O3A	−175.6 (3)	C16—C2—C3—C4B	−4.6 (7)
C5A—C6A—C7A—C8A	4.8 (5)	C9A—C4A—C3—N1	−53.1 (7)
O3A—C7A—C8A—C9A	176.7 (2)	C5A—C4A—C3—N1	129.0 (5)
C6A—C7A—C8A—C9A	−3.6 (4)	C9A—C4A—C3—C2	119.9 (5)
C7A—C8A—C9A—C4A	−0.1 (5)	C5A—C4A—C3—C2	−58.0 (7)
C5A—C4A—C9A—C8A	2.6 (7)	C9A—C4A—C3—C4B	−149 (8)
C3—C4A—C9A—C8A	−175.4 (4)	C5A—C4A—C3—C4B	33 (7)
C9B—C4B—C5B—C6	−3(2)	C5B—C4B—C3—N1	107.3 (15)
C3—C4B—C5B—C6	171.7 (11)	C9B—C4B—C3—N1	−78.2 (17)
C4B—C5B—C6—C7B	2.3 (17)	C5B—C4B—C3—C2	−67.5 (19)
C5B—C6—C7B—C8B	0.3 (14)	C9B—C4B—C3—C2	107.0 (14)
C5B—C6—C7B—O3B	−178.5 (8)	C5B—C4B—C3—C4A	−163 (9)
C17B—O3B—C7B—C6	166.9 (8)	C9B—C4B—C3—C4A	11 (6)
C17B—O3B—C7B—C8B	−11.8 (12)	C3—N1—C10—C15	118.83 (18)
C6—C7B—C8B—C9B	−2.2 (14)	N2—N1—C10—C15	−68.1 (2)
O3B—C7B—C8B—C9B	176.4 (7)	C3—N1—C10—C11	−116.88 (18)
C7B—C8B—C9B—C4B	1.6 (16)	N2—N1—C10—C11	56.22 (19)
C5B—C4B—C9B—C8B	1(2)	N1—C10—C11—C12	179.54 (13)
C3—C4B—C9B—C8B	−173.8 (11)	C15—C10—C11—C12	−55.96 (19)
C3—N1—N2—C1	0.0 (2)	C10—C11—C12—C13	55.8 (2)
C10—N1—N2—C1	−174.16 (16)	C11—C12—C13—C14	−56.0 (2)
N1—N2—C1—C2	0.2 (2)	C12—C13—C14—C15	56.4 (2)
N2—C1—C2—C3	−0.4 (2)	N1—C10—C15—C14	−179.95 (15)
N2—C1—C2—C16	−179.98 (19)	C11—C10—C15—C14	56.3 (2)
N2—N1—C3—C2	−0.20 (19)	C13—C14—C15—C10	−56.4 (2)
C10—N1—C3—C2	173.21 (16)	C3—C2—C16—O1	3.2 (3)
N2—N1—C3—C4A	174.3 (3)	C1—C2—C16—O1	−177.3 (2)
C10—N1—C3—C4A	−12.2 (4)	C3—C2—C16—O2	−176.26 (18)
N2—N1—C3—C4B	−176.1 (6)	C1—C2—C16—O2	3.3 (3)
C10—N1—C3—C4B	−2.7 (6)		

### Hydrogen-bond geometry (Å, °)

**Table:**

*D*—H···*A*	*D*—H	H···*A*	*D*···*A*	*D*—H···*A*
O2—H1O2···O1^i^	0.91	1.73	2.640 (2)	174

Symmetry codes: (i) −*x*, −*y*+1, −*z*+2.
